# A Post-mortem Revealing Two Spleens

**Published:** 1897-07

**Authors:** James M. Richardson

**Affiliations:** House Surgeon, New York College Veterinary Surgeons


### A POST-MORTEM REVEALING TWO SPLEENS.

By James M. Richardson,
HOUSE SURGEON, NEW YORK COLLEGE VETERINARY SURGEONS.

On June 5, 1897, a cat entered the hospital with no other history except that it had not eaten anything for three or four days. By manipulation the case was diagnosed as one of obstruction of the duodenum, and the cat was given warm enemas and castor oil.

On June 11th the animal died in awful agony, and post-mortem showed that what had been taken for an obstruction was a second spleen, which appeared to be perfectly normal. This second spleen was surrounded by a mass of adipose tissue suspended in the mesentery of the small intestines. The misplaced spleen weighed three and one-quarter drachms. There were no abnormal changes to be seen in any of the other organs except a number of small ulcerations throughout the small intestines.

---

Dr. George P. Tucker, of Lincoln, Neb., reports a yearling colt presenting evidence of digestive derangement for a period of from six to eight months, gradually increasing until death from starvation ensued. An autopsy revealed a loop of intestines about twelve feet from the stomach, which had perforated the great omentum and rolled on itself, forming a fibrous ring two inches thick surrounding the loop, so there was but little if any free portion of the omentum. Anterior to the loop the intestines hypertrophied, and at the loop a cavity had formed as large as the stomach. Posteriorly, the intestines were of normal size, with merely a peculiar mottled or pigmented appearance as an additional alteration.

---

Dr. A. Jasme reports the following case: Draft mare while rolling in the box-stall had about two feet of the handle of a broom to enter the rectum, breaking off and being removed by manual force by the owner. The wound was large enough to pass one's hand through the upper part of the rectum, followed by a very œdematous condition of the parts, but under antiseptic injections recovered in three weeks. A recent report from the case would indicate that a fistulous opening still remains, allowing of a slight discharge at times.

---

Dr. Otto Noack reports a case of tetanus in a cat, following castration. Operated upon on June 5th. On the 14th showed stiffness of limbs, tail stiff and carried upward, moving around with great difficulty ; two hours later stiff all over ; ears stiff and drawn together; skin between them drawn in folds; unable to move; could be taken up and put down like a wooden block. If placed on the side no movement could be seen. Later in the day trismus is complete, animal unable to swallow, and the eyes only indicating life at all, death ensuing the same evening.

BURSITIS INTERTUBERCULARIS. Texas pony, black, male, eighteen years of age, 13 hands-high. Sudden soreness after fast driving; at rest the foot is placed backward and resting on toe only; on moving the leg is held in a peculiar position; lower part of the limb is lifted a little from the ground, making at the knee with the upper part an angle of 135 degrees, but no action of the forearm indicating involvement of the flexor brachii; on manipulation from forward backward intense pain at the shoulder is produced, the flexor brachii being the seat and its bursa inflamed. Treatment: sharp liniments were ineffectually employed, afterward repeated blisters of red iodide of mercury, 1 to 3, brought the desired result, with rest of some nine weeks.

